# Deep metagenomic characterization of gut microbial community and function in preeclampsia

**DOI:** 10.3389/fcimb.2022.933523

**Published:** 2022-09-14

**Authors:** Li-Juan Lv, Sheng-Hui Li, Ji-Ying Wen, Guang-Yang Wang, Hui Li, Tian-Wen He, Qing-Bo Lv, Man-Chun Xiao, Hong-Li Duan, Min-Chai Chen, Zhou-Ting Yi, Qiu-Long Yan, Ai-Hua Yin

**Affiliations:** ^1^ Medical Genetic Center, Guangdong Women and Children Hospital, Guangzhou, China; ^2^ Puensum Genetech Institute, Wuhan, China; ^3^ Department of Obstetric, Guangdong Women and Children Hospital, Guangzhou, China; ^4^ Department of Microbiology, College of Basic Medical Sciences, Dalian Medical University, Dalian, China

**Keywords:** preeclampsia, gut microbiome, shotgun metagenomic sequencing, pregnancy, microbial dysbiosis, microbial function

## Abstract

Preeclampsia (PE) is a pregnancy complication characterized by severe hypertension and multiple organ damage. Gut microbiota has been linked to PE by previous amplicon sequencing studies. To resolve the PE gut microbiota in a higher taxonomy resolution, we performed shotgun metagenomic sequencing on the fecal samples from 40 early-onset PE and 37 healthy pregnant women. We recovered 1,750 metagenome-assembled genomes (representing 406 species) from the metagenomic dataset and profiled their abundances. We found that PE gut microbiota had enriched in some species belonging to *Blautia*, *Pauljensenia*, *Ruminococcus*, and *Collinsella* and microbial functions such as the bacitracin/lantibiotics transport system, maltooligosaccharide transport system, multidrug efflux pump, and rhamnose transport system. Conversely, the gut microbiome of healthy pregnant women was enriched in species of *Bacteroides* and *Phocaeicola* and microbial functions including the porphyrin and chlorophyll metabolism, pyridoxal-P biosynthesis, riboflavin metabolism, and folate biosynthesis pathway. PE diagnostic potential of gut microbial biomarkers was developed using both species and function profile data. These results will help to explore the relationships between gut bacteria and PE and provide new insights into PE early warning.

## Introduction

Preeclampsia (PE) is a complex multi-system disease with maternal hypertension and systemic arteriole spasm as basic pathological changes, leading to the second cause of maternal death worldwide (Brown et al., 2018). In addition to severe complications such as eclampsia, cerebral hemorrhage and multiple organ failure, it can also cause fetal intrauterine growth restriction (FGR), fetal distress, premature delivery, stillbirth, and so on (Ahmadian et al., 2020). It is an important factor in increasing perinatal morbidity and mortality, and a serious burden on patients and families. The etiology of PE is still unknown. It was a widely accepted “two-stage theory” that put forward relative or absolute lack of placental perfusion as the key to the pathogenesis mechanism (Redman, 1991). At present, the disease lacks effective prevention and treatment methods. Termination of pregnancy is usually the final and only effective treatment means, resulting in many iatrogenic preterm births. Therefore, effective diagnosis and treatment methods for preeclampsia are urgently needed.

In recent years, many studies have explored the variability of gut microbiota of PE pregnant women in the late trimester (Lv et al., 2019; Wang et al., 2019; Chang et al., 2020; Chen et al., 2020) and linked the imbalance of the gut microbiome to the pathogenesis of PE (Kell and Kenny, 2016). For example, Wang et al. found that the PE subjects had a reduced diversity of microbial alpha diversity and a substantially different bacterial phylum composition compared with the healthy controls. The fecal microbial gene functions related to lipopolysaccharide (LPS) biosynthesis and LPS concentrations of fecal and plasma were higher in the PE group, indicating the pathogenic potential of gut bacterial LPS (Wang et al., 2019). Our previous study revealed that *Blautia, Ruminococcus, Bilophila*, and *Fusobacterium* were significantly enriched in the antepartum gut samples of PE patients compared to healthy controls (Lv et al., 2019). Chang et al. demonstrated that short-chain fatty acids accompanying changes in the gut microbiome could contribute to the development of hypertension in patients with PE (Chang et al., 2020). Also, Chen et al. found that the PE clinical manifestations could be induced in mice models by fecal microbiota transplantation from PE patients, further suggesting the incidence of PE was related to gut microbiota (Chen et al., 2020). However, these amplicon-based studies using the bacterial 16S rRNA genes could only be accurate to the genus level, which made the gut microbiota of PE not fully understood. As a result, no specifically probiotic or pathogenic candidate strains have yet been identified, which limits the exploration of a mechanism or validation of PE in an animal mode.

Herein, we reconstructed a total of 1,750 bacterial MAGs from the shotgun metagenomic sequencing data of the fecal samples. We found that the gut microbiome structure of PE pregnant women was significantly different from that of normal pregnant women. Exactly, 6 members of *Blautia*, 5 members of *Pauljensenia*, 5 members of *Ruminococcus*, and *Fusobacterium ulcerans* were significantly enriched in the feces of PE patients. In contrast, 14 members of Bacteroidaceae, *Akkermansia muciniphila*, and *Bilophila wadsworthia* were significantly reduced in PE pregnant women. The functional signatures of PE-associated gut bacteria were also analyzed, which could provide us with clues to explore their deep pathogenic mechanism. We also tested the diagnostic potential of the gut microbial biomarkers in predicting PE status using a random forest model.

## Methods

### Ethics statement

This cohort study was approved by the Ethics Committee of Guangdong Women and Children Hospital (a tertiary referral hospital specializing in maternal and child health) in southern China, and informed consent was obtained from all participants in accordance with the Declaration of Helsinki (Human, 2001; Idanpaan-Heikkila, 2001).

### Subject recruitment

This study assessed pregnant women receiving pregnancy care at Guangdong Women and Children Hospital. Inclusion criteria were pregnant women with PE and healthy pregnant women who delivered at the same time without pregnancy complications. Exclusion criteria included multiple pregnancies and pregnancy complications such as fetal malformation, gestational diabetes, intrahepatic cholestasis syndrome, or chorioamnionitis. Preexisting clinical disorders, such as diabetes, hypertension, malignant tumors, and infectious diseases were also excluded. In total, 40 pregnant women diagnosed with severe PE (PE group) and 37 normotensive women (HC group) were included in this study. Normal pregnant women in the third trimester were matched with the PE women for age, gestational age, and parity. PE was diagnosed according to the guideline from the American College of Obstetricians and Gynecologists Croke, L. (2019). PE was defined after the 20th week of gestation as blood pressure (BP) at least 140/90 mmHg on two occasions for at least 4h with previously normal BP; proteinuria at least 300 mg/24-h urine or more collection. In the absence of proteinuria, new onset of any of the following: platelet count less than 100,000 ×10^9^/L; serum creatinine concentration more than 1.1 mg/dL or a doubling in the absence of other renal diseases; elevated blood concentrations of liver transaminases to twice normal concentration; pulmonary edema and cerebral or visual symptoms. The clinical biochemistry parameters of all participants were measured according to the methods described in our previous study (Lv et al., 2019). The detailed information of the clinical and phenotypic characteristics of the participants are summarized in **Table 1**.

**Table 1:** Basic characteristics of the preeclampsia patients and healthy controls included in this study.

	Preeclampsia (n = 40)	Healthy control (n = 37)	*P*-value
**Age (y)**	31.4 ± 5.0	30.0 ± 4.1	0.178
**Gestational age (day)**	246 ± 25	273 ± 11	3.8x10^-8^
**Pre-pregnancy BMI (kg/m^2^)**	21.7 ± 3.3	20.5 ± 3.2	0.190
**Current BMI**	26.6 ± 3.9	26.0 ± 3.0	0.529
**SBP (mmHg)**	147.6 ± 22.0	114.2 ± 10.6	1.6x10^-8^
**DBP (mmHg)**	92.8 ± 15.1	68.6 ± 8.9	2.2x10^-8^
**GLU (mmol/L)**	4.41 ± 0.89	4.27 ± 0.25	0.592
**% HbA1c**	5.15 ± 0.73	4.99 ± 0.45	0.435

The data for preeclampsia patients and healthy controls were presented as mean ± SD. *P*-values were calculated by Student’s *t*-test. SBP, systolic blood pressure; DBP, diastolic blood pressure; GLU, fasting blood glucose.

### Sample collection, DNA extraction and shotgun metagenomic sequencing

All fecal samples were collected in the third trimester. Fecal samples were collected with sterile feces collection containers and stored rapidly at -80̊ until use. The total DNA of fecal samples (170mg per sample) was extracted using the Tiangen fecal DNA extraction kit (Tiangen, China) according to the manufacturer’s instructions. DNA concentration and purity were determined by NanoDrop2000 and Qubit 4.0. Total DNA was fragmented using Covaris M220 (Gene Company Limited, China). The sequencing libraries were prepared using the TruSeq DNA sample prep kit (Illumina, United States). Paired-end shotgun metagenomic sequencing was performed based on the Illumina NovaSeq platform (Novogene Co. Ltd, China), which generated 2 × 150 bp paired-end reads for further analyses. Initial base calling of the metagenomic dataset was performed based on the system default parameters under the sequencing platform.

### Bioinformatic analyses

Raw metagenomic sequencing reads were processed for quality controls using fastp (version 0.23.0) (Chen et al., 2018) with parameters “-q 20 -u 30 -n 5 -y -Y 30 -g -x -l 90 -w 20”. Low quality (>45 bases with quality score<20, or >5 ‘N’ bases), low complexity, and adapter-containing reads were removed, and the remaining reads were trimmed at the tails for low quality (<Q20) or ‘N’ bases. Human genomic reads were removed *via* mapping to the reference human genome (GRCh38) using Bowtie2 (version 2.4.4) (Langmead and Salzberg, 2012) with default parameters in the “–end-to-end –fast” mode.

High-quality clean reads were used for *de novo* assembly *via* MEGAHIT (v1.2.9) (Li et al., 2015) with a broad range of k-mer sizes (–k-list 21,41,61,81,101,121,141). Assembled contigs (minimum length threshold 2,000 bp) were binned using MetaBAT 2 (version 2.12.1) (Kang et al., 2019) with default parameters. Only raw bins with a total size >200 kbp were kept for further analyses. The sequencing depth of bins was calculated by mapping the high-quality reads back to the bins with Bowtie2 (version 2.4.4) (Langmead and Salzberg, 2012) based on the formula: $Depth=\frac{Total\_mapped\_reads\;\times \;Average\_length\_of\_reads}{Total\_length\_of\_the\_bin}$ .Taxonomic classification of the bins was realized based on the GTDB-Tk toolkit (v1.4.0) (Chaumeil et al., 2019) *via* assigning the sequences of each bin to the Genome Taxonomy Database (version r95) (Parks et al., 2020) with default parameters. The taxonomic name of the bins was manually modified to accord with traditional nomenclatures following the national center for biotechnology information (NCBI) taxonomy. To increase the genomic completeness of bins, in each sample, raw bins were merged if they had approximately equal sequencing depth ( ± 10% between each other) and G+C content ( ± 0.02 between each other) and had an identical taxonomic assignment at the species level.

The quality of MAGs was estimated with CheckM (v1.1.3) (Parks et al., 2015) using the *lineage_wf* workflow. The definition of high- and medium-quality MAGs was based on the minimum information about a metagenome-assembled genome (MIMAG) standards (Bowers et al., 2017) (high: ≥90% completeness and<5% contamination; medium: ≥70% completeness and<5% contamination). And the quality score was defined as “QS = completeness – 5 × contamination”, following Parks et al. (2017). All high- and medium-quality MAGs with quality score >60 were clustered at the nucleotide level by dRep (v2.2.3) (Olm et al., 2017), for which the MAGs sharing >95% average nucleotide identity (ANI) were treated as redundancies. A MAG with the highest QS was chosen as the representative MAG of each cluster (referred to as “species”). Finally, the high-quality sequencing reads of each sample were mapped against the nonredundant MAG catalog using Bowtie2 (version 2.4.4) (Langmead and Salzberg, 2012) (default parameters in the “–end-to-end –fast” mode) to generate the relative abundance of these MAGs. For the taxonomic profiles at the phylum, class, order, family, and genera levels, we summed the relative abundance of MAGs from the same taxon to yield the abundance of that taxon. A phylogenetic tree of MAGs was built using PhyloPhlAn (version 3.0.58) (Asnicar et al., 2020) and visualized in iTOL (v4) (Letunic and Bork, 2019).

*Ab initio* microbial genes were identified from the contigs of each MAG using Prodigal (v2.6.3) (Hyatt et al., 2010). The KEGG (The Kyoto Encyclopedia of Genes and Genomes) database (downloaded in June 2021) was used for functional annotation of genes using the BlastKOALA algorithm (Kanehisa et al., 2016). Each protein was assigned a KEGG orthologue (KO) on the basis of the best-hit gene in the database (e-value<1e-10 and covering >50% of the protein length). KOs were assigned into pathways or modules based on the KEGG website (https://www.kegg.jp). The abundance of a functional category (KO, pathway, or module) was calculated from the summation of the relative abundance of its corresponding genes.

### Statistical analyses

Statistical analyses were implemented on the R 4.0.1 platform. Principal coordinate analysis (PCoA) was performed with the R *vegan* package, based on the Bray-Curtis dissimilarly, and visualized *via* the R *ade4* package. Permutational multivariate analysis of variance (PERMANOVA, also known as *adonis* analysis) was realized with the R vegan package, and the *adonis P*-value was generated based on 1,000 permutations. Mantel test was performed using the R *ade4* package. Random forest models were analyzed with the R *randomForest* package (1,000 trees). The performance of the predictive model was evaluated using receiver operator characteristic (ROC) analysis which was realized with R *pROC* package. The *q* value was used to evaluate the false discovery rate (FDR) for correction of multiple comparisons and was calculated using the R *fdrtool* package based on the Benjamin-Hochberg method (Strimmer, 2008).

## Results

### Subjects and construction of metagenome-assembled genomes

To uncover the gut microbial characteristics of PE, we recruited 40 pregnant women with early-onset PE and 37 healthy pregnant women and collected their fecal samples for metagenomic analyses. Patients and controls were matched in their age, pre-pregnancy BMI, and current BMI (**Table 1**). On average, the gestational age at the sampling time point of the PE patients was approximately 4 weeks less than the healthy controls (35w+1d vs. 39w, *p*<0.001). For the clinical parameters, the systolic blood pressure (SBP) and diastolic blood pressure (DBP) of patients were significantly higher than those of healthy controls, consistent with the clinical feature of PE (Lv et al., 2019). The fasting glucose level and %HbA1c (glycosylated hemoglobin) were approached between two cohorts.

We performed deep shotgun metagenomic sequencing of 77 fecal samples, obtaining a total of 781.0 Gb of high-quality non-human data (on average, 10.1 Gb per sample; **Table S1**) for analysis. To investigate the gut microbiome at the species and strain levels, we reconstructed a total of 1,750 MAGs from the fecal metagenomes using a recently developed method (Almeida et al., 2019). The genome size of MAGs ranged from 1.07 to 6.63 Mb (on average, 2.51 Mb), with N50 length ranging from 4.2 to 106.1 kb (on average, 66.0 kb; **Table S2**). Based on the MIMAG standard (Bowers et al., 2017), 59.5% (1,042/1,750) MAGs were high-quality microbial genomes (completeness ≥90%, contamination<5%), 37.9% (663/1,750) MAGs were medium-quality genomes (completeness 70-90%, contamination<5%, and quality score ≥60), while the remaining 2.6% (45/1,750) MAGs were low-quality genomes with completeness 50-70% and contamination 5%.

**Figure 1:**
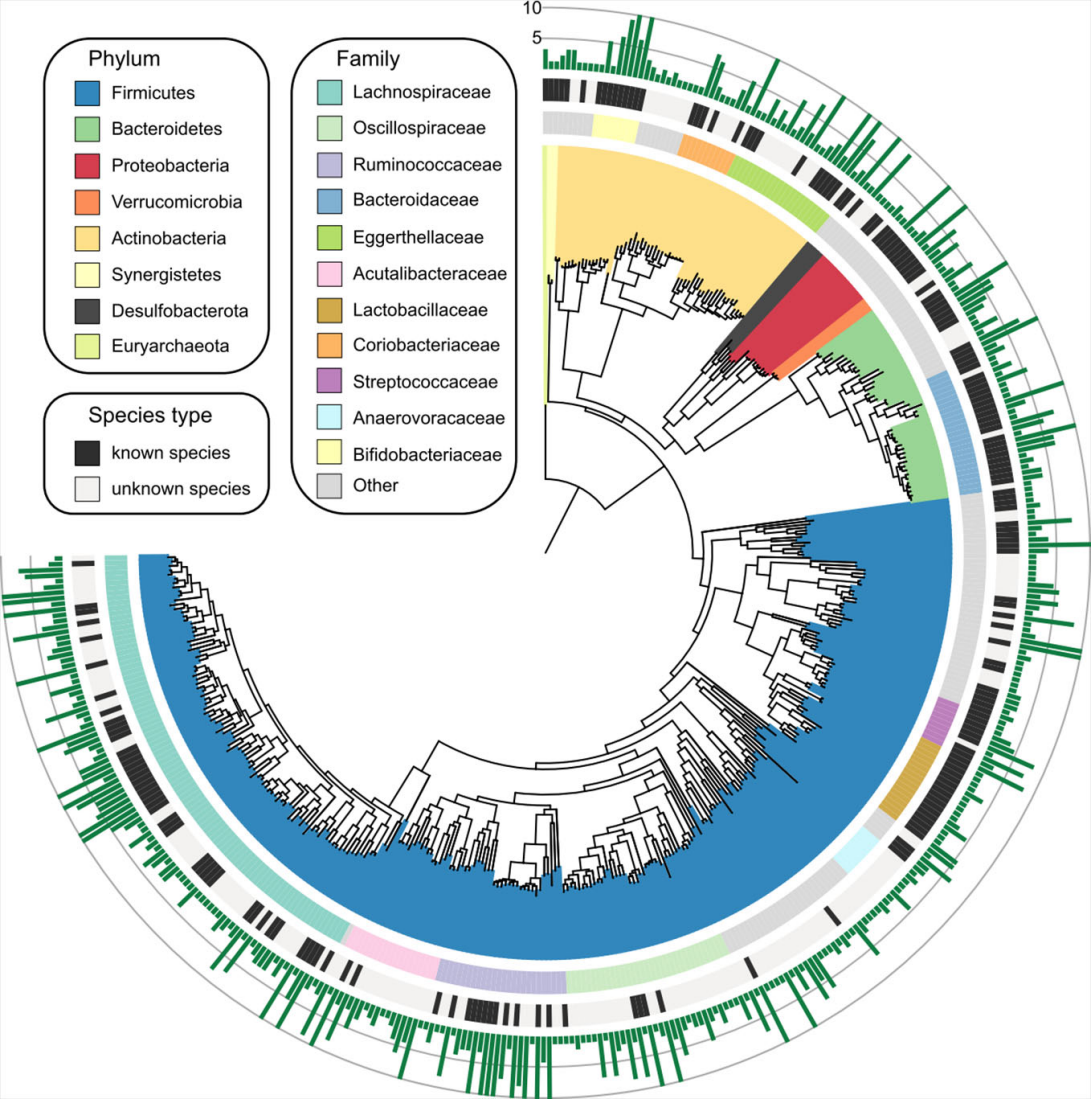
Genome-wide representation of 406 prokaryotic species. The innermost circle: phylogenetic tree analysis of 406 species based on their genome sequences. The colors in the tree indicate the phylum-level taxonomic assignment of the species. Circle 2: taxonomic assignment of the species at the family level. Circle 3: the species that could be assigned into known species are labeled in black, and the remaining species are uncultivated. Outermost circle, the number of MAGs for each species. Species with more than 10 MAGs were cropped into 10 for visual clarity, and the detailed information of all species was listed in **Table S3**.

Based on the genomic similarity threshold (ANI 95%) at the prokaryotic species level (Jain et al., 2018), the 1,750 MAGs were clustered into 406 species-level bins (referred to as “species” hereinafter). For each species, we chose a MAG as the representation of the species based on the highest quality. The phylogenetic tree and the summary of taxonomic assignment of 406 species were shown in **Figure 1**, and the detailed information of them was provided in **Table S3**. The species were assigned into 7 bacterial phyla (consisting of 405 species) and 1 archaeal phylum (1 species). The major phyla of all species included Firmicutes (consisting of 282 species), Actinobacteria (58 species), and Bacteroidetes (43 species). At the family level, Lachnospiraceae (93 species), Oscillospiraceae (33 species), Ruminococcaceae (26 species), Bacteroidaceae (25 species), Eggerthellaceae (22 species), and Acutalibacteraceae (Parks et al., 2015) were dominant families of the species catalogue. Notably, only 47.2% (192/406) of the species could be assigned into known species, whereas the others were uncultivated species in the human gut.

### Gut microbiome diversity and overall structure in relation to PE

**Figure 2:**
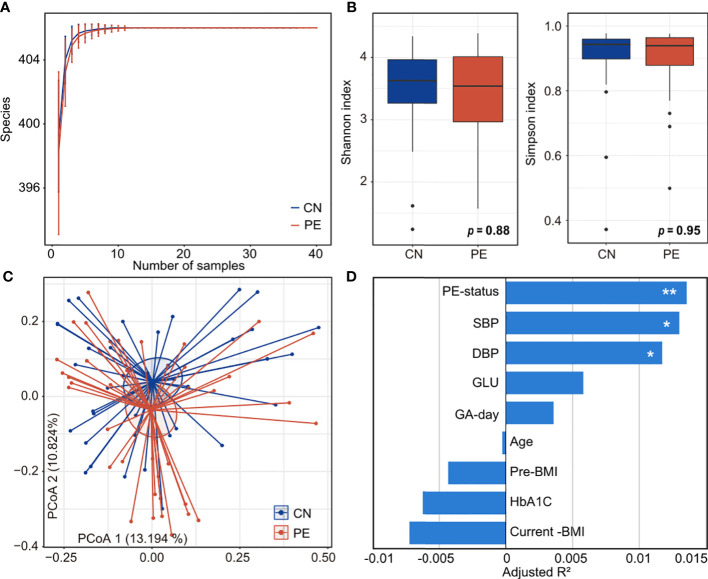
Comparison of microbial diversity and structure in PE patients and healthy controls. **(A)** Rarefaction curve analysis of the number of observed species in two groups. The number of species in different groups is calculated based on a randomly selected specific number of samples with 30 replacements, and the median and quartile values are shown. **(B)** Boxplot shows the Shannon and Simpson diversity indexes of gut microbiota that differ among the two groups. Boxes represent the interquartile range between the first and third quartiles and median (internal line); whiskers denote the lowest and highest values within 1.5 times the range of the first and third quartiles, respectively; and nodes represent outliers beyond the whiskers. **(C)** PCoA analysis of Bray-Curtis distance based on the composition of gut microbiota, revealing the separation between the PE patients and healthy controls. The location of samples (represented by nodes) in the first two principal coordinates is shown. Lines connect samples in the same group, and circles cover samples near the center of gravity for each group. **(D)** PERMANOVA analysis showing the effect size of phenotype indexes that contribute to the variance of the overall gut microbiome. Bar plots indicate the explained variation (adjusted R^2^) of each factor. *Adonis* test: *, permutated *p*<0.05; **, permutated *p*<0.01. SBP, systolic blood pressure; DBP, diastolic blood pressure; GLU, fasting blood glucose; GA-day, gestational age (day); Pre-BMI, pre-pregnancy body mass index.

To explore the alteration of diversity and structure in the PE gut microbiome, we profiled the composition of 406 gut species of 77 fecal metagenomes and compared it between the PE patients and healthy controls. Rarefaction analysis showed that, under nearly ten samples in PE and control group, the rarefaction curve is approximately saturated (**Figure 2A**). We then used the Shannon diversity index and Simpson index to assess the microbial richness and evenness at the species level. No significant difference was found in these indexes between the two cohorts (**Figure 2B**), suggesting that the PE doesn’t impact the microbial diversity in the gut microbiome. Next, PCoA analysis based on the Bray-Curtis distance revealed a visible alteration of the overall gut microbial structure between PE patients and the control group (**Figure 2C**). In addition, PERMANOVA analysis using the distance matrix showed that the PE-status explained 1.4% of microbial variations (permutated *p*=0.005). As a comparison, the subjects’ clinical parameters, including age, pre-pregnancy, current BMI, gestational age, fasting glucose, and %HbA1c, didn’t significantly impact the overall microbial variations (**Figure 2D**). Also, two PE-related clinical indexes, SBP and DBP, significantly acted on the gut microbiota. These results suggested that PE patients undergo profound changes in their gut microbiome.

### Identification of PE-associated microbial families and species

**Figure 3:**
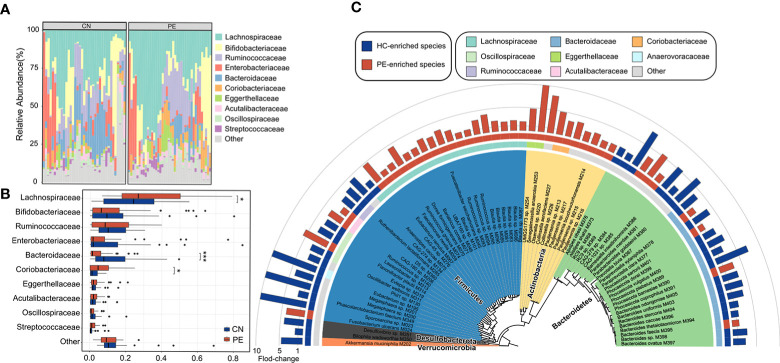
Family- and species-level alteration of the gut microbiota in PE patients. **(A)** Composition of gut microbiota at the family level. **(B)** Boxplot shows the dominant families of the gut microbiota when compared between the PE patients and healthy controls. **(C)** Detailed information of 74 species that differed in abundance between the gut microbiota of PE patients and healthy controls. Innermost circle, phylogenetic tree analysis of 74 PE-associated species based on their genome sequences. The colors in the tree indicate the phylum-level taxonomic assignment of the species. Circle 2: taxonomic assignment of the species at the family level. Circle 3: enrichment direction of the species. Red and blue label the species that enriched in the PE patients and controls, respectively. Outermost circle: barplot shows the fold changes of species abundance in PE patients compared with that in healthy subjects.

We compared the gut microbial composition of PE patients and healthy controls at the family level. Lachnospiraceae, Bifidobacteriaceae, Ruminococcaceae, Enterobacteriaceae, Bacteroidaceae, Coriobacteriaceae, and Eggerthellaceae were the most dominant families, with an accumulative relative abundance of over 81.3% in all analyzed samples (**Figure 3A**). Of these families, Lachnospiraceae (*q*=0.026) and Coriobacteriaceae (*q*=0.048) were significantly enriched in the gut microbiota of PE patients compared with those of the healthy controls, while Bacteroidaceae (*q*<0.001) were marked depleted in the PE patients (**Figure 3B**).

At the species level, 74 of 406 species were identified as PE-associated species with significant differences in relative abundance between two cohorts (Wilcoxon rank-sum test, *p*<0.05; corresponding to FDR 12.6%; **Table S4**). 39 of these species were more abundant in the gut microbiota of PE patients, while 35 were enriched in the healthy controls. The PE-enriched species included 7 members of *Blautia* (unknown at the species level), 5 members of *Pauljensenia* (containing *P. bouchesdurhonensis* and 4 uncultivated species), 5 members of *Ruminococcus* (containing R. gnavus and 4 uncultivated species), *Fusobacterium ulcerans*, and others (**Figure 3C**). Consistent with the findings at the family level, the control-enriched species included 14 members of Bacteroidaceae, containing 7 *Bacteroides* spp., 4 *Phocaeicola* spp., a *Prevotella bivia*, a *Paraprevotella clara*, and a *Barnesiella intestinihominis* species. Also, an *Akkermansia muciniphila* species and a *Bilophila wadsworthia* species were enriched in the gut microbiota of healthy controls. Of note, several phylogenetically closely related species showed an opposite tendency between PE patients and healthy controls, such as *Phocaeicola coprophilus/sartorii* vs. *Phocaeicola plebeius/vulgatus/barnesiae/dorei* and *Paraprevotella xylaniphila* vs. *Paraprevotella clara* (**Figure 3C**), suggesting that their difference in functions may relate to PE.

### Identification of PE-associated functional signatures

**Figure 4:**
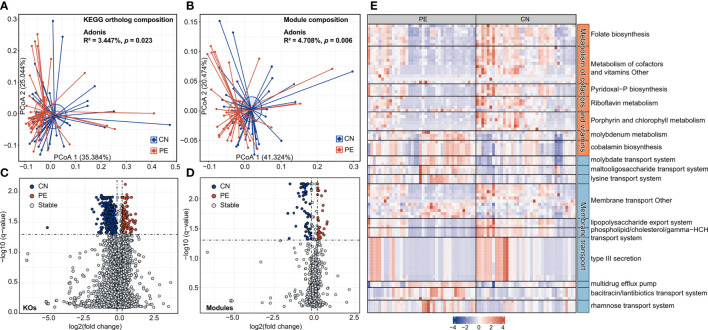
Functional comparison of the gut microbiome. **(A, B)** PCoA analysis of Bray-Curtis distance based on the functional composition of gut microbiota at the KO **(A)** and module **(B)** levels. The location of samples (represented by nodes) in the first two principal coordinates is shown. Lines connect samples in the same group, and circles cover samples near the center of gravity for each group. **(C, D)** Volcano plots show the fold change vs. *q*-values for the KOs **(C)** and modules **(D)**. The X-axis shows the ratio (log2 transformed) of function abundance in PE patients compared with that in healthy controls. The Y-axis shows the *q*-value (-log10 transformed) of a function. The functions that were enriched in PE and control subjects are shown in red and blue points, respectively. **(E)** A heatmap showing the abundance of PE-associated KOs involved in the metabolism of cofactors and vitamins and membrane transport pathways. Each column represents an individual and each row represents a KO. The pathway categories of the KOs are grouped and the detailed information of these KOs are shown in **Table S5**.

**Figure 5:**
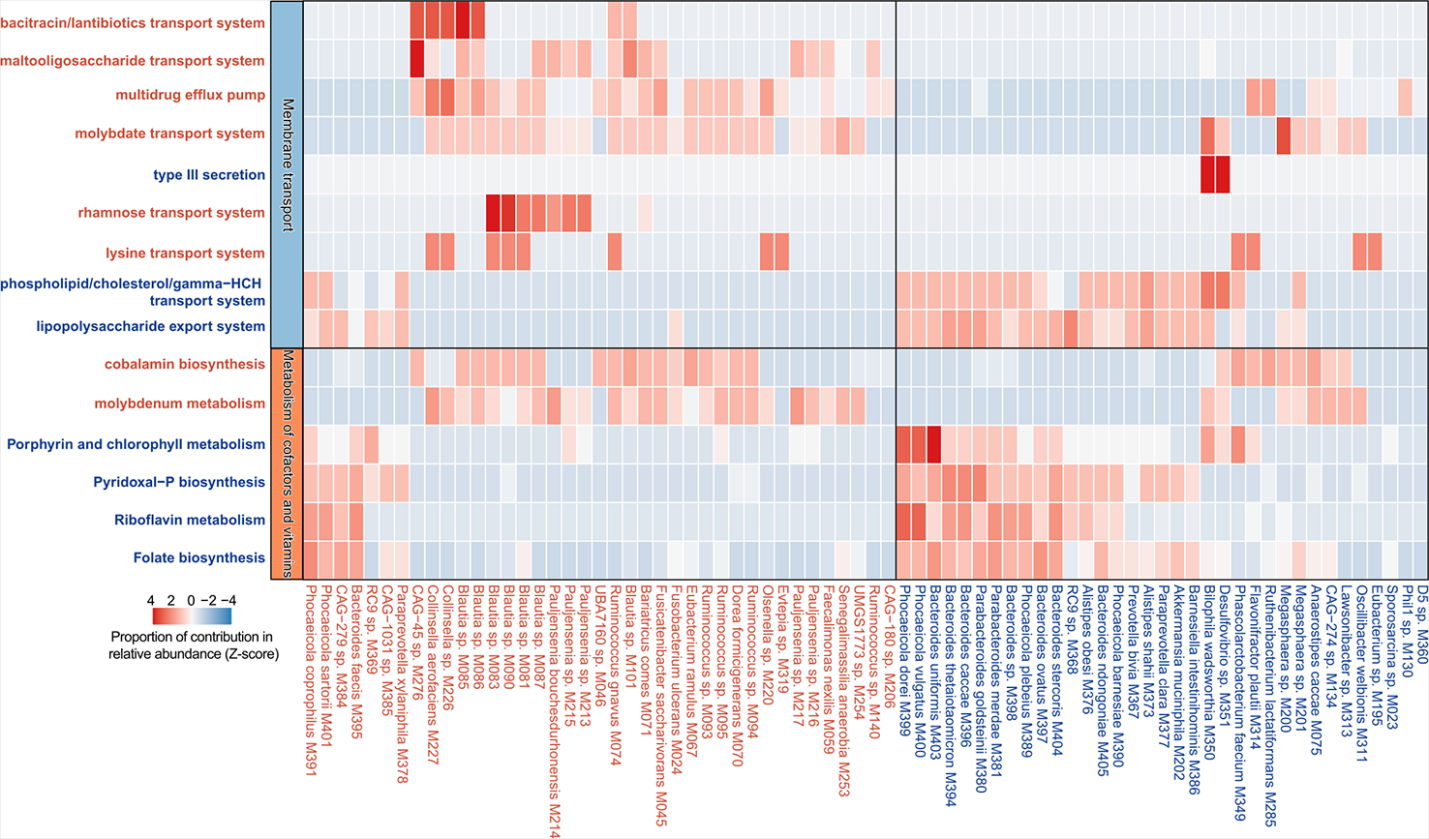
Contribution of species on the abundance of functional categories. A heatmap showing the proportional contribution of 74 PE-associated species on the abundance of 15 functional categories shown in **Figure 4E**. The species that were enriched in the gut microbiota of PE patients and healthy controls are labeled in red and blue, respectively.

In order to describe the functional characteristics of the PE microbiome, we annotated the functions of 406 species *via* the KEGG database (Kanehisa et al., 2017) and profiled the functions of all fecal samples. A total of 7,399 KOs and 679 KEGG modules were involved. Similar to the aforementioned taxonomic composition, the overall functional capacity of PE patients also changed considerably compared to that of the healthy controls (effect size of KOs = 3.4%, PERMANOVA *p*=0.023; effect size of modules = 4.7%, PERMANOVA *p*=0.006; **Figures 4A, B**). There were significant differences in the relative abundance of 618 KEGG orthologs (KOs) and 107 modules between the patient and control group (Wilcoxon rank-sum test, *q*<0.05; **Figures 4C, D**). 115 and 503 KOs were enriched in the gut microbiota of PE patients and healthy controls, respectively. The HC-enriched KOs were frequently involved in the enzymes of core pathways including energy metabolism, amino acid metabolism, carbohydrate metabolism, glycan biosynthesis and metabolism, and lipid metabolism; while the PE-enriched KOs usually participated in functions such as genetic information processing, membrane transport, and signaling and cellular processes (**Table S5**). We especially focused on the PE-associated KOs involved in the membrane transport and cofactors/vitamins metabolism pathways. The PE-enriched KOs belonging to the cofactors and vitamins metabolism pathway were mainly composed of cobalamin biosynthesis (containing 5 KOs) and molybdenum metabolism (3 KOs), on the other hand, the HC-enriched KOs of cofactors/vitamins metabolism pathway were related to folate biosynthesis (containing 7 KOs), porphyrin and chlorophyll metabolism (6 KOs), pyridoxal-P (vitamin B6) biosynthesis (4 KOs), riboflavin metabolism (4 KOs), and others (**Figure 4E**). The PE-enriched KOs of the membrane transport pathway contained the transport systems of rhamnose (containing 4 KOs), bacitracin/lantibiotics (4 KOs), molybdate (3 KOs), maltooligosaccharide (3 KOs), lysine (3 KOs), and multidrug efflux pump (2 KOs), while the HC-enriched KOs of membrane transport pathway were dominated by type III secretion (containing 14 KOs), lipopolysaccharide export system (3 KOs), and phospholipid/cholesterol/gamma-HCH transport system (3 KOs). Moreover, we quantified the contribution of 74 PE-associated species on these functions, based on the functional configuration information of the species (**Figure 5**). This analysis connected the PE-associated species and functions and led to several notable findings. For example, the enzymes of the bacitracin/lantibiotics transport system were mainly encoded by PE-enriched *Collinsella* and *Blautia* species, and the enzymes of rhamnose transport system were encoded by PE-enriched *Blautia* and *Pauljensenia* species. In contrast, functions of porphyrin and chlorophyll metabolism, pyridoxal-P biosynthesis, riboflavin metabolism, and folate biosynthesis were dominantly encoded by the members of *Bacteroides* and *Phocaeicola* that were both more abundant in the gut microbiota of HC subjects.

### Diagnostic potential of gut microbial biomarkers

**Figure 6:**
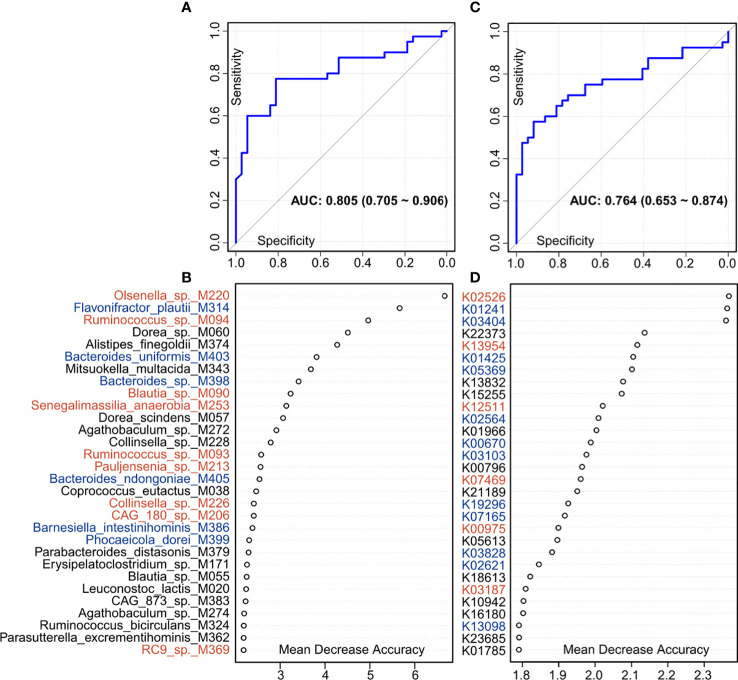
Classification of PE status by the relative abundances of gut microbial biomarkers. **(A, C),** ROC analysis for classification of PE patients and healthy controls by species **(A)** and KO **(C)** profiles, assessed by AUC. **(B, D)** The 30 most discriminant species **(B)** and KOs **(D)** in the model classifying patients and controls. Colors represent enrichment in PE patients (red) or healthy controls (blue).

Finally, we used the random forest regression model to evaluate the performance of the gut microbiome in identifying PE status. A random forest model based on the gut species profile obtained the discriminatory power of the area under the receiver operating characteristic curve (AUC) of 0.805 (95% confidence interval [CI] 0.705–0.906; **Figure 6A**). Several PE-enriched species, including *Olsenella* sp. M220, *Ruminococcus* sp. M094, *Blautia* sp. M090, and *Senegalimassilia anaerobia* M253, as well as several control-enriched species, including *Flavonifractor plautii* M314, *Bacteroides uniformis* M403, and *Bacteroides* sp. M398, featured the highest score for the discrimination of PE patients and healthy controls (**Figure 6B**). Likewise, a model based on the KO profile obtained the discriminatory power of AUC of 0.764 (95% CI 0.653–0.874; **Figure 6C**). Several PE-enriched KOs, including K02526 (2-keto-3-deoxygluconate permease), K13954 (alcohol dehydrogenase), and K12511 (tight adherence protein), as well as several control-enriched KOs, including K01241 (AMP nucleosidase), K03404 (magnesium chelatase), K01425 (glutaminase), and K05369 (dihydrobiliverdin/ferredoxin oxidoreductase), featured the highest discrimination score in the model (**Figure 6D**).

## Discussion

Although a series of 16S rRNA gene-based studies have uncovered the relationships between gut microbiota and PE (Lv et al., 2019; Wang et al., 2019; Chang et al., 2020; Chen et al., 2020), deep metagenomic characterization of the PE gut microbiota has still not been reported. In this study, 406 species (as identified from representative MAGs), including 214 human gut uncultivated species indicated the advantages of whole metagenome methods and extended the existing database. More accurate PE-associated bacterial species and functional characteristics of the PE microbiome were identified. For example, PE-enriched multiple strains of genus *Collinsella*, *Pauljensenia*, *Ruminococcus* and *Blautia*, and HC-enriched multiple strains of genus *Bacteroides*, *Phocaeicola*, and *Parabacteroides*, were the potential key strains and typical representative among them (**Figure 3C**). The enzymes of the bacitracin/lantibiotics transport system, the rhamnose transport system, and the maltooligosaccharide transport system encoded by PE-enriched *Blautia* and *Collinsella* species, the enzymes of the phospholipid/cholesterol/gamma-HCH transport system, the lipopolysaccharide export system, the porphyrin and chlorophyll metabolism, the Pyridoxal-P biosynthesis, the riboflavin metabolism, and the folate biosynthesis mainly encoded by HC-enriched *Bacteroides* and *Parabacteroides* species, were the importantly PE-associated functional signatures (**Figure 5**). The well-defined gut microbial biomarkers of PE status will contribute to the PE’s early warning.

Eight genera were found enriched in antenatal PE feces in our previous study, of which *Blautia* and *Ruminococcus2* represented the major variances in PE microbiomes and were associated with most PE-enriched functional modules, suggesting their central role in the PE microbiome (Lv et al., 2019). Herein, higher sequencing accuracy reached the species level, uncovering 6 members of *Blautia* and 5 members of *Ruminococcus* (*R. gnavus* and 4 uncultivated species). *Blautia* is a genus containing over 20 anaerobic species that are widely present in the mammalian gut (Liu et al., 2021). As a dominant genus in the intestinal microbiota, the increased abundance of genera *Blautia* (Eckburg et al., 2005; Round and Mazmanian, 2009; Human Microbiome Project, 2012) and *Ruminococcus* was positively correlated with irritable bowel syndrome (Rajilic-Stojanovic et al., 2011), ulcerative colitis (Nishino et al., 2018), gestational diabetes mellitus (Crusell et al., 2018), preeclampsia (Lv et al., 2019; Liu et al., 2021; Miao et al., 2021), pregestational weight, hyperlipidemia (Miao et al., 2021), obesity and dyslipidemia, which involved lipid metabolism, glycosyl-transferases, biotin metabolism, and the oxidative-phosphorylation pathway (Miao et al., 2021). Maternal blood pressure and liver enzyme levels were positively correlated to *Ruminococcus* (Lv et al., 2019). *R. gnavus* was a definite species of PE enriched *Ruminococcus* genus. *R. gnavus* were associated with advanced coronary artery diseases (CAD) (Toya et al., 2020), inflammatory bowel disease (Hall et al., 2017), and obesity (Petriz et al., 2014), which could secrete a complex polysaccharide that potently induces inflammatory cytokine (TNF-α) secretion by dendritic cells (Henke et al., 2019).

Previous studies have revealed the enrichment of LPS in peripheral blood of PE women (Lv et al., 2019) which may produce a severe inflammatory response leading to the development of PE (Sanchez-Aranguren et al., 2014). It causes insufficient infiltration of placental trophoblast cells, abnormal spiral artery remodeling, and placental ischemia and hypoxia (Riley and Nelson, 2010). Chen et al. transplanted fecal bacteria from PE patients and found that mice showed PE-like symptoms, increased peripheral blood LPS and increased placental inflammation (Chen et al., 2020). PE enriched *Fusobacterium* contained lipopolysaccharide, and could promote immune abnormalities, stimulate inflammatory response, and damage intestinal barrier function (Lv et al., 2019). Its thermal killing effect on chorionic cells mainly depended on the LPS activity (Barak et al., 2007), which was positively correlated with blood pressure in pregnant women. Due to the increased accuracy of shotgun metagenomic sequencing, *Fusobacterium ulcerans* was a representative species in the PE-enriched gut microbiota in this study, which had confirmed the cytotoxic effect and may contribute to the pathogenesis of tropical ulcers (Adriaans and Garelick, 1989; Veraldi et al., 2021).

*Collinsella* genus is enriched in the PE gut and belongs to the family Coriobacteriaceae of the phylum Actinobacteria. Members of the family Coriobacteriaceae are frequently considered as pathobionts and can affect metabolism by altering intestinal cholesterol absorption, decreasing glycogenesis in the liver, and increasing triglyceride synthesis. *Collinsella* increases gut permeability by reducing the expression of tight junction proteins ZO-1 (Chen et al., 2016). Its abundance has been associated with type 2 diabetes (T2D), rheumatoid arthritis (Chen et al., 2016), and cholesterol metabolism (Chen et al., 2016). *Collinsella* contributes to the pathogenesis in rheumatoid arthritis by increasing gut permeability, reducing the expression of tight junction proteins in epithelial cells, and inducing the expression of IL-17 cytokines (Chen et al., 2016), which may result in the pathologic effects by recruitment of neutrophils and activation of NF-kB signaling (Derrien et al., 2009). The increased gut permeability leads to leakage of bacteria and endotoxins from the gut lumen to the mesenteric lymph nodes and portal circulation, which will stimulate peritoneal, intestinal and hepatic macrophages (Kim et al., 2006) to release numerous inducer cytokines of PE (Lv et al., 2019; Chen et al., 2020). We found that intestinal fatty acid binding protein, a biomarker of the integrity of the tight junction barrier of peripheral blood intestinal epithelial cells in PE pregnant women, was higher than in the control group, indicating that the intestinal barrier was impaired in PE pregnant women (Lv et al., 2019).

Furthermore, Th17 cellular immunity is dominant in PE (Molvarec et al., 2015; Zhang et al., 2018; Eghbal-Fard et al., 2019). The imbalance of Th17/Treg cells dominated by Th17 cellular immunity can cause exaggerated systemic inflammation (Quinn et al., 2011; Toldi et al., 2015), endothelial dysfunction and placental dysangiogenesis, which are the pathogenesis of PE (Vargas-Rojas et al., 2016). The Th17 response is mainly characterized by the production of inflammatory cytokines, including IL‐17A (Eghbal-Fard et al., 2019). IL‐17 stimulates fibroblasts, MQs, DCs, endothelial cells and epithelial cells to generate multiple pro‐inflammatory mediators, resulting in further inflammatory immune response (Kleinewietfeld and Hafler, 2013). Moreover, IL‐17 also induces placental and renal oxidative stress and placental vascular dysfunction resulting in the development of hypertension (Cornelius and Lamarca, 2014). Therefore, we speculated that Collinsella might be related to the increase of IL-17 in PE, but the specific mechanism needs to be further clarified. Overall, these results suggest that many PE-enriched bacteria were related to the damage of the intestinal barrier, the promotion of immune abnormalities, the activation of inflammatory response, and the metabolic abnormalities of PE patients, and may be involved in the pathogenesis of PE through the entero-placental pathway.

Another strength of this whole metagenome-based study is the precise analysis of functional genes of microorganisms. PE-enriched KOs commonly participated in functions such as genetic information processing, membrane transport, and signaling and cellular processes (**Table S5**). The membrane transport systems partially mediated the interactions between the gut microbiota and host cells (Konishi et al., 2015). Membrane transport is an indispensable step for importing/exporting essential molecules into/out cells and exists in all tissues of living organisms. The breakdown of transport systems could induce various diseases (Konishi et al., 2015). For example, the changed expression levels of Peptide transporter 1 transporters (PEPT1) lead to the enhanced signaling pathways associated with inflammatory reactions (Konishi et al., 2015). In this study, the PE-enriched KOs of the membrane transport pathway contained the transport systems of rhamnose (containing 4 KOs), bacitracin/lantibiotics (4 KOs), molybdate (3 KOs), maltooligosaccharide (3 KOs), lysine (3 KOs), and multidrug efflux pump (2 KOs). The enzymes of the rhamnose transport system were encoded by PE-enriched *Blautia* and *Pauljensenia* species. The bacitracin/lantibiotics transport system was mainly encoded by PE-enriched *Collinsella* and *Blautia* species (**Figure 5**). Lantibiotics are produced by many Gram-positive bacteria and kill susceptible cells primarily through membrane pore formation with a strong and wide spectrum of antimicrobial activity against Gram-positive bacteria (Willey and van der Donk, 2007; Lobo-Ruiz and Tulla-Puche, 2018). Some members of the intestinal microbiome, such as *Ruminocossus gnavis* and *Blautia obeum*, can produce lantibiotics, which may cause intestinal microbiota dysbiosis (Guinane et al., 2016; Weiss and Hennet, 2017; Garcia-Gutierrez et al., 2019). The bacteriocins production capability of *Blautia* leads to the reduced intestinal colonization of some pathogenic bacteria (Liu et al., 2021). Yonezawa et al. demonstrated that lantibiotic bacteriocins produced in oral bacteria may be one of the causative factors of intestinal microbiota dysbiosis (Yonezawa et al., 2021). In addition, PE-enriched bacteria, such as *Desulfovibrio* (Korenblum et al., 2005), *Bacteroides* (Guinane et al., 2016), and *Eubacterium* (Le Blay et al., 2007), could be inhibited by the bacitracin/lantibiotics produced by gut microbiota. These suggest that bacitracin/lantibiotics produced by *Collinsella* and *Blautia* species may interfere with the composition of the gut microbiota of PE patients.

The enzymes of the multidrug efflux pump (MDR) and molybdate transport system were mainly encoded by PE-enriched *Blautia* and *Collinsella* species, which were associated with bacteria virulence. For example, *Pseudomonas aeruginosa* improved bacterial competition advantage by promoting molybdate acquisition, and the molybdate transport system is critical to bacterial virulence (Wang et al., 2021). Multidrug efflux pumps are ancient elements encoded in microbial chromosomes. In addition to being mainly related to the pathogenicity of bacteria (Piddock, 2006; Blanco et al., 2016), they are also associated with drug resistance (Piddock, 2006). The expression of MDR efflux pumps is induced by host-produced compounds which can play a role in the virulence of bacterial pathogens (Piddock, 2006). Notably, this efflux pump is involved as well in the bacterial capability for biofilms formation (Baugh et al., 2014; Wang et al., 2021).

In addition, we also found HC-enriched KOs frequently involved in the enzymes of core pathways including energy metabolism, amino acid metabolism, carbohydrate metabolism, glycan biosynthesis and metabolism, and lipid metabolism. More impressively, the enzymes of the pyridoxal-P biosynthesis, Riboflavin metabolism, and folate biosynthesis were more encoded by HC-enriched *Bacteroides caccae*, *Bacteroides thetaiotaomicron*, *Bacteroides uniformis*, *Phocaeicola vulgatus* etc. (**Figure 5**). Generally, the insufficient synthesis of these vitamins is a potential PE pathogenic factor (Brophy and Siiteri, 1975; Wacker et al., 2000; Singh et al., 2015). The phospholipid/cholesterol/gamma-HCH transport system of HC-enriched *Bacteroides* are important anti-inflammatory bacteria in the gut and could decrease cholesterol levels in the plasma and benefit health (Yazdanyar et al., 2011). In this study, the decreased *Phocaeicola* (former name: *Bacteroides*) *vulgatus* was worth noting, which was not only involved in the folate biosynthesis, etc., but also could fight against LPS-induced acute intestinal injury and DSS-induced colitis (Li et al., 2021; Wang et al., 2022). After all, these are the potential risk factors of PE (Ahmadian et al., 2020).

## Conclusion

Deep shotgun metagenomic sequencing significantly improved the accuracy of species identification within the PE gut microbiota. This led to a more comprehensive and in-depth exploration of microbial functional genes and potential pathogenic mechanisms. We observed that *Blautia*, *Ruminococcus, Collinsella*, *Bacteroides*, and *Phocaeicola* might relate to the PE occurrence or development. Moreover, the bacitracin/lantibiotics transport system, maltooligosaccharide transport system, multidrug efflux pump, rhamnose transport system, porphyrin and chlorophyll metabolism, pyridoxal-P biosynthesis, riboflavin metabolism, and folate biosynthesis pathway were also significant changes in the PE gut microbiota. The diagnostic biomarkers of gut microbiota were beneficial to the early warning of PE. The future study might therefore focus on isolating the PE-associated gut bacteria and applying them to the verification experiments to elucidate these abovementioned pathogeneses.

## Data availability statement

The datasets presented in this study can be found in online repositories. The names of the repository/repositories and accession number(s) can be found in the article/**Supplementary Material**.

## Ethics statement

The studies involving human participants were reviewed and approved by Ethics Committee of Guangdong Women and Children Hospital. The patients/participants provided their written informed consent to participate in this study.

## Author contributions

A-HY contributed to the conception of the work and manuscript guidance. Q-LY contributed to the experiment and manuscript guidance. L-JL mainly carried out the cohort research performance, data analysis, and drafted the manuscript. S-HL and Q-BL were responsible for statistics, analysis, and functional annotation of bioinformatics data. G-YW, T-WH, and M-CX participated in the implementation of the experiment. J-YW, HL, H-LD, M-CC, and Z-TY were responsible for sample storage and fellow-up of the cohort. All the participants provided approval for publication of the content, agreed to be accountable for all aspects of the work in ensuring that questions related to the accuracy or integrity of any part of the work are appropriately investigated and resolved.

## Funding

This work was supported by Guangdong Basic and Applied Basic Research Foundation (2019A1515110389) and Medical Scientific Research Foundation of Guangdong Province (B2019013).

## Conflict of interest

The authors declare that the research was conducted in the absence of any commercial or financial relationships that could be construed as a potential conflict of interest.

## Publisher’s note

All claims expressed in this article are solely those of the authors and do not necessarily represent those of their affiliated organizations, or those of the publisher, the editors and the reviewers. Any product that may be evaluated in this article, or claim that may be made by its manufacturer, is not guaranteed or endorsed by the publisher.
